# N_2_ Gas Flushing Limits the Rise of Antibiotic-Resistant Bacteria in Bovine Raw Milk during Cold Storage

**DOI:** 10.3389/fmicb.2017.00655

**Published:** 2017-04-19

**Authors:** Patricia Munsch-Alatossava, Susanna Jääskeläinen, Tapani Alatossava, Jean-Pierrre Gauchi

**Affiliations:** ^1^Department of Food and Environmental Sciences, University of Helsinki, University of HelsinkiFinland; ^2^MaIAGE, INRA, Université Paris-SaclayJouy en Josas, France

**Keywords:** antibiotic resistance (AR), raw milk, cold storage, N_2_ gas, lactoperoxidase system, Ryan-Einot-Gabriel-Welsch test

## Abstract

Antibiotic resistance has been noted to be a major and increasing human health issue. Cold storage of raw milk promotes the thriving of psychrotrophic/psychrotolerant bacteria, which are well known for their ability to produce enzymes that are frequently heat stable. However, these bacteria also carry antibiotic resistance (AR) features. In places, where no cold chain facilities are available and despite existing recommendations numerous adulterants, including antibiotics, are added to raw milk. Previously, N_2_ gas flushing showed real potential for hindering bacterial growth in raw milk at a storage temperature ranging from 6 to 25°C. Here, the ability of N_2_ gas (N) to tackle antibiotic- resistant bacteria was tested and compared to that of the activated lactoperoxidase system (HT) for three raw milk samples that were stored at 6°C for 7 days. To that end, the mesophiles and psychrotrophs that were resistant to gentamycin (G), ceftazidime (Ce), levofloxacin (L), and trimethoprim-sulfamethoxazole (TS) were enumerated. For the log_10_ ratio (which is defined as the bacterial counts from a certain condition divided by the counts on the corresponding control), classical Analyses of Variance (ANOVA) was performed, followed by a mean comparison with the Ryan-Einot-Gabriel-Welsch multiple range test (REGWQ). If the storage “time” factor was the major determinant of the recorded effects, cold storage alone or in combination with HT or with N promoted a sample-dependent response in consideration of the AR levels. The efficiency of N in limiting the increase in AR was highest for fresh raw milk and was judged to be equivalent to that of HT for one sample and superior to that of HT for the two other samples; moreover, compared to HT, N seemed to favor a more diverse community at 6°C that was less heavily loaded with antibiotic multi-resistance features. Our results imply that N_2_ gas flushing could strengthen cold storage of raw milk by tackling the bacterial spoilage potential while simultaneously hindering the increase of bacteria carrying antibiotic resistance/multi-resistance features.

## Introduction

The statement is unanimous among the highest international authorities: the increasing prevalence of antibiotic-resistant bacteria constitutes one of the most serious threats to human health; both the WHO and FAO have issued action plans to tackle antibiotic resistance (AR), which is perceived as a global and increasing threat (WHO, 2015; FAO, 2016). In Europe, it is estimated that AR already induces extra health costs and productivity losses amounting to at least 1,500 million euros, and it leads to 25,000 deaths annually (WHO, 2015). Although, the European Union already placed a ban on using antibiotics as growth promoters in 2006, some countries still sell more antibiotics for animal use than for treating humans. Due to the simultaneous presence of spoilage microorganisms in food materials, of food-borne pathogens and antibiotic-resistant bacteria, food production systems face great challenges. The warning that “the resistance problem in human medicine will not be solved if there is a constant influx of resistance genes into the human microflora via the food chain” was already made earlier (Teuber, 1999). Food has been identified as one main direct vehicle for the transmission of antibiotic-resistant bacteria from animals to humans and AR genes that are carried by zoonotic bacteria (Perreten et al., 1997; SØrum and L'Abbé Lund, 2002; Aquilanti et al., 2007; Garofalo et al., 2007; Wang et al., 2012; Rolain, 2013; ECDC/EFSA/EMA, 2015). Wichmann et al. (2014) also demonstrated that the cow microbiome was a significant reservoir of AR genes; however, the scientific community still remains divided regarding the contribution of antibiotic use on food production, despite the fact that more evidence points to the anthropogenic causes of increasing AR in both human or environmental microbiomes (Forslund et al., 2014).

Although milk is considered to be sterile when it is secreted from a healthy udder, various contamination sources increase its bacterial level. Depending on the production area, the farm type and the milk handling practices, the types and levels of bacteria vary greatly in raw milk (Chambers, 2002; Frank and Hassan, 2002). In developed countries, recommendations state that the bacterial load in raw milk should not exceed 10^5^ cfu/ml and 3 × 10^5^ cfu/ml “total bacterial counts” in the farm and dairy tanks, respectively. These counts are determined under mesophilic incubation conditions (3 d at 30°C) (Anonymous, 2004); there are no recommendations concerning AR in raw milk. With regards to antibiotics (ABs), the dairy industry states that raw milk should not contain AB residues (which could compromise the growth of the starter strains that form the bases of yogurt or cheese production). If ABs are present in milk, then the farmer is not allowed to sell this milk, which is then used to feed cattle, with the risk that the bacteria that have become resistant may enter the food chain (Pereira et al., 2014; Andremont, 2015).

Two options are recommended for the preservation of raw milk: either cold storage (at approximately 4°C/below 6°C) or the activation of the lactoperoxidase system (HT) when economical or technical constraints prevent the use of cooling facilities (FAO, 1991, 2005). When raw milk is cold-stored, psychrotrophic/psychrotolerant bacteria take over within a few days and promote the spoilage of the raw milk due to the production of varied and mostly heat-stable enzymes (Chambers, 2002). On the other side, in the absence of a cold chain infrastructure, many adulterating substances, including antibiotics, are used to prevent excessive bacterial growth in raw milk (Bari et al., 2015; Botelho et al., 2015).

During earlier analyses of bacterial isolates that were involved in raw milk spoilage, resistance to several classes of antibiotics (or multi-resistance) seemed to increase during the cold storage of raw milk (Munsch-Alatossava and Alatossava, 2006, 2007). Further investigations revealed that psychrotrophic bacterial populations exhibited higher AR levels compared to their corresponding mesophilic populations; most importantly, based on their AR profiles, distinct bacterial populations succeeded one another during cold storage; strangely enough, the AR was highest at the time when the “total bacterial counts” had reached approximately 10^5^ cfu/ml; after that, the relative AR level was lower but the milk was spoiled (Munsch-Alatossava et al., 2012a).

The limitations of raw milk cold storage, together with the observation that cold storage seems to promote an increase in AR (Munsch-Alatossava and Alatossava, 2007; Munsch-Alatossava et al., 2012a,b), motivated research efforts to attempt to control bacterial growth in raw milk more effectively. Two studies examined the use of N_2_ gas to prevent bacterial growth in raw milk that was kept in a “closed system” (Murray et al., 1983; Dechemi et al., 2005). By considering an “open system,” both culture-dependent investigations and DNA barcoding studies revealed that no pathogen, no spoilage bacteria or any anaerobe was clearly advantaged by the N_2_ gas flushing treatment when it was applied to raw milk at the laboratory scale, despite the fact that 10^4^- fold lower bacterial counts differentiated the N_2_-flushed from non-flushed cold-stored raw milk samples (Munsch-Alatossava et al., 2010a,b; Gschwendtner et al., 2016). Recently, the efficiency of N_2_ gas flushing and the activated lactoperoxidase system in controlling bacterial growth in raw milk samples stored at 15 and 25°C was compared; overall, the gas treatment showed a time-limited effect that was comparable to the effect of the activated lactoperoxidase system (Munsch-Alatossava et al., 2016). Because the N_2_ gas flushing inhibited the mesophilic and psychrotrophic populations in cold-stored raw milk, we undertook an investigation as to whether the N_2_ gas-based treatment could also inhibit the growth of antibiotic-resistant bacteria in raw milk stored at 6°C, and its efficiency was compared to that of the activated lactoperoxidase system for both mesophiles and psychrotrophs. Consequently, bacteria that were resistant to gentamycin (G), ceftazidime (Ce), levofloxacine (L), and trimethoprim-sulfamethoxazole (TS) were enumerated from three cold-stored raw milk samples while they were subjected to the following three conditions: the application of N_2_ gas flushing, the activation of the lactoperoxidase system (HT) and no additional treatment as the control (C). For the statistical treatment of the data, we defined the ratio as the amounts of colonies that were recovered in the presence of one antibiotic type divided by the number of colonies enumerated from the corresponding control. During the whole study, the decimal logarithm of the ratio, i.e., the log_10_ (ratio), was analyzed with the Ryan-Einot-Gabriel-Welsch (REGWQ) multiple range test (Hsu, 1996).

## Materials and methods

### Materials and treatment of raw milk samples

Three bovine raw milk samples (S1, S2, and S3), which represent commingled lorry milk delivered to Helsinki Dairy Ltd. in Helsinki (Finland) in November and December 2015, were subjected. After the raw milk arrived, 100 ml of each sample were added to 250 ml sterile bottles and placed on a multi-place magnetic stirrer (Variomag, Oberschleißheim, Germany). The bottles were partially immersed in a refrigerated water bath (MGW Lauda MS/2), which allowed, with the help of an immersion thermostat, a constant temperature to be maintained (Munsch-Alatossava et al., 2010a). The raw milk samples were mixed continuously at 220 rpm and kept at 6 ± 0.1°C for 7 days. The activation of the lactoperoxidase system (HT) consisted in the addition of both hydrogen peroxide and thiocyanate at 10 ppm each (FAO, 2005); the source of H_2_O_2_ was a 30% H_2_O_2_ solution (Perdrogen ^R^ 30 Gew%, Riedel de Häen, Seelze, Germany). The thiocyanate anion SCN^−^ took the form of NaSCN (Sigma-Aldrich, Steinheim, Germany) and a 1% (w/v) stock solution was sterile-filtered and cold- stored until use. The N_2_ gas (AGA Ltd, Riihimäki, Finland) was 99.999% pure and the flow rate for the continuous N_2_ gas flushing treatments (N) was adjusted to 120 ml/min (Munsch-Alatossava et al., 2010a). The description of the conditions (which were applied to every sample) is as follows: C, control; HT, H_2_O_2_+ SCN^−^ (for the activated lactoperoxidase system); and N, N_2_ gas flushing.

### Microbiological analyses

Antibiotic resistance was quantified by enumerating bacterial colonies on agar plates that contained one of each of the considered antibiotics (ABs) (each one is representative of one AB class) and by comparing their levels to the corresponding “no AB” (no antibiotics) control. The analyses were performed at day 0 (shortly after the samples were received) and after 3 and 7 days of cold storage. All the bacterial counts were determined mostly from triplicate if not duplicate platings on Mueller-Hinton agar (Lab M, Ltd, Lancashire, UK). The antimicrobial agents [gentamycin (aminoglycosides), ceftazidime (β-lactams, cephems), levofloxacin (quinolones) and trimethoprim-sulfamethoxazole (at a ratio of 1/19, a folate pathway inhibitor) (Sigma-Aldrich, Steinheim, Germany)] which are abbreviated G, Ce, L, and TS, respectively, were added to the agar in accordance with the EUCAST guidelines (EUCAST, 2000). The AB solutions were freshly prepared by dissolving the powders into the following solvents: MilliQ water for G, 0.1M phosphate buffer (pH 7) for Ce, 0.1M NaOH for L, 0.1M lactic acid for T, and 95% ethanol for S (EUCAST, 2000). With the exception of S, all the AB solutions were filter-sterilized prior to their addition to adequately cooled agar. The final AB concentrations in the agar plates were 16 mg/L for G, 32 mg/L for Ce, 8 mg/L for L and 8 mg/L trimethoprim together with 152 mg/L sulfamethoxazole for TS. These concentrations correspond to 4-fold of the MIC for G, Ce and L, or 2-fold the MIC for TS, as indicated by EUCAST for pseudomonads (EUCAST, 2010). All the agar plates were stored overnight at 4°C and protected from light before use. Following the analyses performed as described before (Munsch-Alatossava et al., 2012a,b), the plates were incubated under aerobic conditions for 2–3 days at 30°C, or for 10 days at 7°C to enumerate the “total” bacterial mesophilic and psychrotrophic counts, respectively.

### Statistical analyses

Statistical analyses were performed independently for every raw milk sample. To compare the efficiency of the treatments, the analyses employed the ratio that was defined as “the counts obtained in the presence of one of the four antibiotic (AB) types divided by the counts enumerated on the corresponding control.” To overcome the variability of microbiological data (excessively low or high bacterial counts), the decimal logarithm of the ratio [log_10_ (ratio)] was analyzed. When the ratio denominator of the bacterial counts was “zero,” “zero” was replaced with “one.” To overcome the situations in which the ratios were equal to zero and to allow for log transformation, 0.0001 was added to all the values. The statistical analyses relied on a classical Analysis of Variance (ANOVA) followed by a multiple comparison of the means with the Ryan-Einot-Gabriel-Welsch test (REGWQ) (Hsu, 1996) with an alpha risk of 0.05, as applied previously (Munsch-Alatossava et al., 2016). All the calculations were performed with SAS/STAT software version 9.4/ GLM procedure (SAS Institute, NC, USA). Low vs. high values of the log_10_(ratio) indicate that the bacterial populations were strongly or poorly controlled by the applied treatments. The statistical analyses determined the influence of the following three factors: (1) the “time” or duration of cold storage (which addressed the trends in the counts between days 0 and 3, and between days 0 and 7) visualized by two levels “day 3” and “day 7”; (2) the ”treatment type” of either non-treated raw milk, which accounts for the control (C), or where the lactoperoxidase system was activated (HT), or for N_2_-flushed milk (N); and (3) the “AB type,” which considered the response to the following five conditions: the absence of any antibiotic (no AB), or the presence of one of the antibiotics (whether G, Ce, L, or TS). Moreover, two-factor and three-factor interactions were evaluated for the log_10_ (ratio).

## Results

### Impact of the treatments on the raw milk pH

For all three raw milk samples (S1, S2, and S3) in which the lactoperoxidase (HT) was activated, the pH values that were measured after 7 days of cold storage were nearly equal to the initial pH values (Table 1). In addition, the 7-day-N_2_ gas flushing (N) did not greatly alter the initial pH values of the samples, given that the drop ranged between 0.02 and 0.14. As expected, the non-treated raw milk samples (C) showed the largest pH decreases (from 0.3 to 0.5 unit) at the end of the storage period (Table 1).

**Table 1:** **pH values for raw milk samples (S1 to S3), determined at initial and final stages (after 7d storage at 6°C), for the three conditions: non treated raw milk (C), activated lactoperoxidase system (HT), and N_**2**_ gas flushed (N) milk**.

**Raw milk samples**	**Initial pH**	**C**	**Final pH HT**	***N***
S1	6.81	6.35	6.81	6.73
S2	6.74	6.46	6.76	6.60
S3	6.75	6.25	6.78	6.73

### Growth trend in resistant bacteria during cold storage

#### Bacterial levels in non-treated raw milk (C)

**Figure 1:**
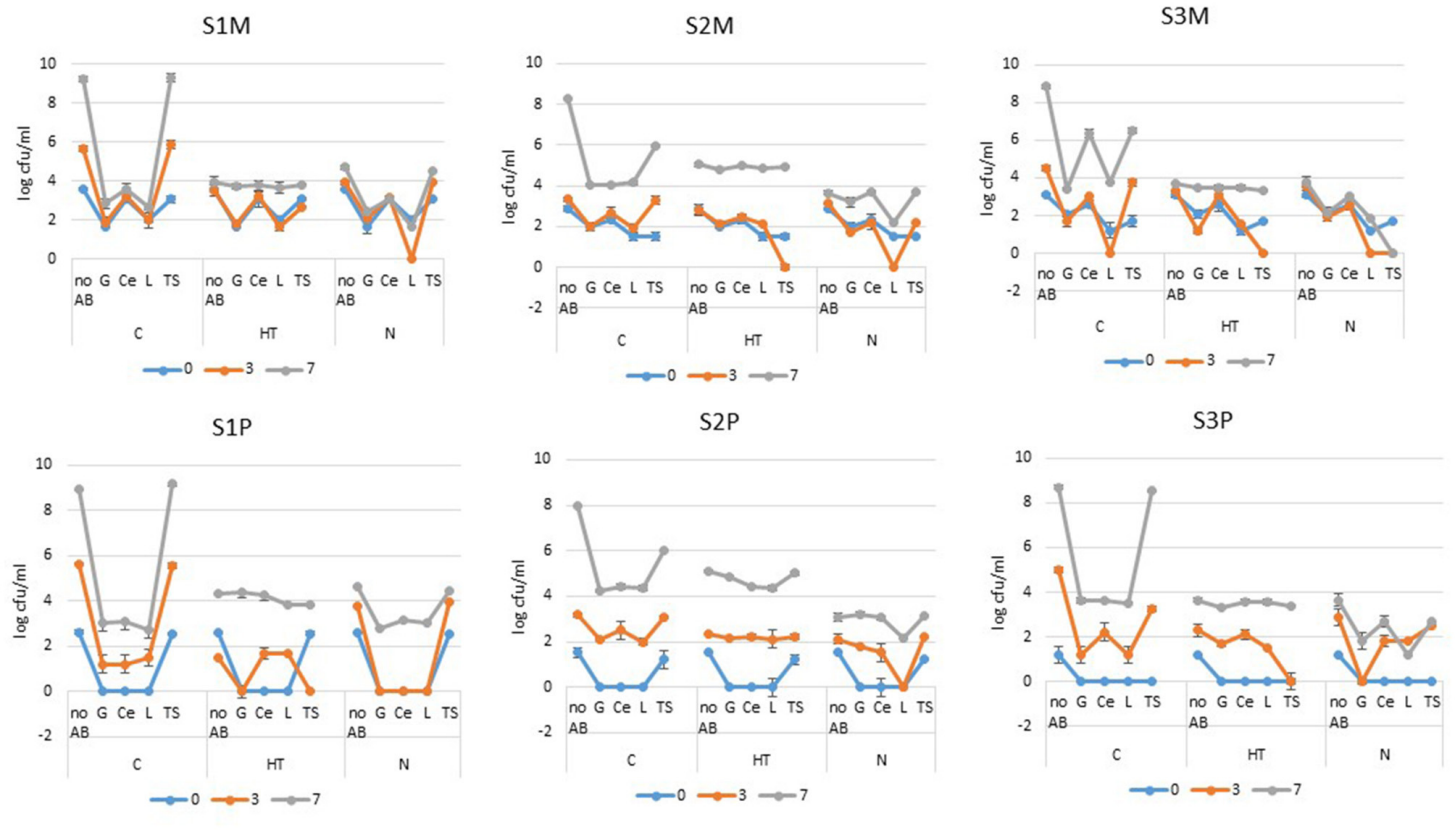
**Mesophilic (M) and psychrotrophic (P) bacterial counts (expressed in log cfu/ml) from raw milk samples S1, S2, and S3 that were stored for 7 days at 6°C (C), cold-stored while the lactoperoxidase system was activated (HT) or cold-stored while flushed with N_**2**_(N)**. The colonies were enumerated on Mueller-Hinton agar plates that contained no antibiotics (“no AB”) or one of the following ABs: G (gentamycin), Ce (ceftazidime), L (levofloxacin) and TS (trimethoprim-sulfamethoxazole) (the error bars correspond to standard deviations).

At the beginning of the storage period, all three samples presented initial “total” mesophilic counts (S1M, S2M, and S3M) that ranged between 734 and 3,700 cfu/ml (2.86 and 3.57 log-units) (Figure 1). Despite having rather similar initial counts, the samples revealed different bacterial growth kinetics for the “no AB” condition after 3 days of cold storage given that the counts were below 10^4^ cfu/ml for S2M, below the limit (3 × 10^5^ cfu/ml or 5.48 log units) for S3M and close to 10^6^ for S1M; the additional 4 days of cold storage promoted 3 to 5 log-units of additional increases in the counts for all three samples (Figure 1). At the initial stage, all the samples showed detectable levels of mesophilic-resistant bacteria, which were highest with Ce and TS for S1M and highest with Ce for both S2M and S3M. After 3 days of cold storage for all the samples, the TS-resistant mesophiles had increased and even reached an equal level to that of “no AB” for S1M and S2M; the G-, Ce-, and L-resistant bacterial levels largely remained constant out of a 1 log-unit drop for L-resistant bacteria for S3M (Figure 1). After 7 days of cold storage, the G-, Ce-, and L-resistant counts had increased moderately for S1M compared to that of “no AB”; between 3 and 7 days, the resistant bacterial counts mostly increased for S2M and S3M. For G and L, the levels were equivalent for both S2M and S3M (slightly below or approximately 4 log-units). The Ce-resistant counts were also at this level for S2M, but they were considerably higher for S3M for which the levels were “equal” to that of the TS-resistant bacteria. For all three samples, at the end of the storage period, the levels of resistant mesophiles were below 3 × 10^5^ cfu/ml (5.48 log units) for the controls (C), with the exception of TS-resistant bacteria, and of Ce-resistant bacteria in addition for S3M (Figure 1).

Psychrotrophs that were resistant to G, Ce, and L were initially below the detection levels for all three samples, whereas TS-resistant bacteria were recovered at levels equivalent to the “no AB” counts for S1P and S2P (Figure 1). In contrast to the mesophiles, for which “day 3” was rather equivalent to “day 0,” resistant psychrotrophs increased notably in all three samples (Figure 1). The augmentation continued until the end of the storage period; by that time, the Ce-resistant counts had increased by approximately 3-fold for G, Ce and L for S1P, over 4-fold for S2P and over 3-fold for S3P. However, out of the TS-resistant psychrotrophs that culminated in 10^9^, 10^6^, or above 10^8^ cfu/ml for S1P, S2P, and S3P, respectively, all the other psychrotrophic counts remained below 3 × 10^5^ cfu/ml at day 7 (Figure 1).

#### Bacterial levels in raw milk under the activated lactoperoxidase system (HT)

After 3 days under cold storage, the “no AB” condition revealed that the mesophilic counts remained unchanged at approximately 3 log-units for S2M and they were slightly above the initial levels for S1M and S3M. Compared to the corresponding controls (C), the counts for “no AB” were approximately 2 log-units below the levels recorded for S1M and S3M, whereas no drop was recorded for S2M. After 7 days, HT had provoked a large inhibition of approximately 5 log-units of bacterial growth for S1M and S3M compared to approximately 3 log-units lower counts for S2M (Figure 1). For resistant mesophiles, rather minor changes occurred during the first 3 days: this finding is well illustrated for S1M under the activated lactoperoxidase system (HT), when in the presence of G, Ce and L, the mesophilic counts remained unchanged during the first 3 days and were at the level of the corresponding control (C); for S1M, day 7 was primarily characterized by a moderate increase in Ce- and TS-resistant bacteria, a greater increase in G- and L-resistant bacteria, and all the counts, including those of “no AB,” were at approximately 4 log-units. If for S1M, the counts for “no AB” and TS were clearly lower for HT compared to the control (C), the G- and L-resistant bacteria were moderately higher compared to the levels of the corresponding control (C) (Figure 1). For S2M at day 3, the counts for “no AB,” G-, Ce-, and L- resistant bacteria were at approximately their initial levels. However, the activated lactoperoxidase (HT) system triggered a notable change at day 3 concerning TS-resistant bacteria, which were reduced to a non-detectable level (Figure 1). Day 7 was characterized by a large increase (of approximately 5 log-units) of bacteria that were resistant to TS, which were only one log-unit lower than the level attained by TS-resistant bacteria for C. Although the counts that were enumerated on the G-, Ce-, and L-plates had increased by approximately 3 log-units, they were still below 3 × 10^5^ cfu/ml, but they exceeded the levels recovered for the corresponding control (C) by approximately 1 log-unit (Figure 1). For S3M, HT promoted a drop in G- and TS-resistant mesophiles (to an undetectable level in the latter case) at the intermediate storage time; with the exception of the mesophilic counts recovered on Ce containing agar (for which the counts remained fairly equivalent during the course of the experiment), the bacteria that were resistant to G, L and TS greatly increased between 3 and 7 days for S3M, and they were approximately 3.5 log-units, which corresponded to the levels of G- and L-resistant bacteria that were found for the control (C) (Figure 1).

At the initial stage under the activated lactoperoxidase (HT), G-, Ce-, and L-resistant psychrotrophs were below the detectable levels in all three samples (Figure 1). For TS-resistant bacteria, the situation was most contrasted given that the counts were below the detectable level for S3P, and at the “no AB” level for S1P and S2P. Among the psychrotrophic populations, the sharpness of the increase in resistant bacteria was highest for S2P (Figure 1). For S1, contrary to the mesophiles, the psychrotrophs (S1P) showed lower counts at day 3 than the “no AB” condition as compared to day 0. During the 3-day storage period, the psychrotrophs that were resistant to G, Ce and L increased in all the samples with the exception of G for S1P. TS still promoted the most distinctive response. Regarding the increase in resistant counts for S2P, the counts remained at an undetectable level for S3P and showed a considerable drop in the counts between days 0 and 3 for S1P (to an undetectable level) (Figure 1). The additional 4 days of cold storage promoted a considerable increase in the AR for all three samples; irrespective of the AB type, the resistant psychrotrophs reached more or less approximately the same level as the counts from the “no AB” condition. However, HT prevented the excessive growth of TS-resistant psychrotrophs, especially for S1P and S3P (Figure 1).

#### Bacterial levels in N_2_-flushed raw milk (N)

For the N_2_ gas flushing treatment (N) at day 7, the “no AB” condition revealed a moderate increase in bacterial counts that was equivalent to HT for S3M, slightly above that of HT for S1M, and below HT for S2M, but also below the levels encountered for the controls (C) (Figure 1). The 7- day-cold storage period, together with the N_2_ gas flushing, promoted fairly minor changes in AR- resistant mesophiles considering S1M and S3M, compared to S2M (Figure 1). After 3 days of cold storage, quite similar patterns in the responses were observed for G-, Ce-, and L-resistant bacteria for all three raw milk samples. The level of G-resistance was lower than that of Ce, whereas L-resistant bacteria were reduced to non-detectable levels in all three samples. The TS-resistant bacteria either increased moderately for S1M and S2M, or were reduced to a non-detectable level during the first 3 days of cold storage for S3M (Figure 1). After 7 days, the highest counts were observed in TS for S1M, with Ce and TS in the case of S2M. The maximum inhibitory effect was recorded for S3M, because all of the resistant bacteria were equivalent to or below 10^3^ cfu/ml; the resistant mesophiles were largely less numerous under N compared to HT (Figure 1).

Compared to the mesophiles, more changes were observed for the psychrotrophs for all three samples under the N_2_ flushing treatment for the “no AB” condition; at day 0, no colonies that were resistant to G, Ce or L were detected in S1P, S2P, and S3P. If TS-resistant bacteria were present in S1P and S2P (equivalent to levels of “no AB”), none were detected for S3P (Figure 1). After 3 days of storage, G-, Ce-, or L-resistant colonies remained under the detectable level for S1P; the same was observed in L-resistant bacteria for S2P and for G-resistant bacteria for S3P (Figure 1). By contrast, the TS-resistant colonies increased over the 3 days for all 3 samples, and they reached the levels that were observed for the “no AB” condition. At the end of the storage period, S1P and S2P showed a more homogeneous picture (for 3 ABs out of 4), whereas for S3P, the resistant populations were at approximately the same level with G and L on one side, and with C and TS on the other side. The resistant psychrotrophic counts from N were mostly below the AR levels recorded from the corresponding controls (C) or from the activated lactoperoxidase system (HT) (Figure 1).

### Statistical analyses

#### Impact of the storage “time” factor on bacterial growth

**Figure 2:**
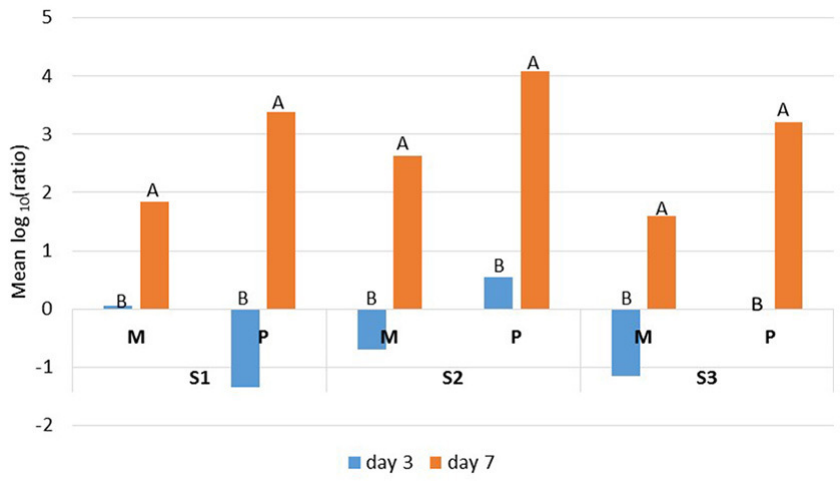
**REGWQ results depicting the impact of either 3 or 7 days of storage at 6°C on mesophilic (M) and psychrotrophic (P) populations that were recovered from the raw milk samples S1, S2, and S3**. Means with different letters indicate significant differences (alpha risk = 0.05).

The Ryan-Einot-Gabriel-Welsch (REGWQ) multiple range test was applied to the variable log_10_ (ratio), which enabled us to quantify the changes that occurred during the storage period. The results showed a decrease in the bacterial counts, depicted by “negative” values, which were indicative of an effective control for mesophiles from samples S2 and S3 and for psychrotrophs from sample S1 at day 3 only (Figure 2). For all three samples, the mean log_10_ (ratio) values were considerably and significantly higher at day 7 compared to day 3, which confirmed that the additional four days of cold storage favored the proliferation of mesophilic and psychrotrophic bacterial populations. However, the increase in psychrotrophs (+4.73/S1, +3.54/S2, and +3.22/S3) supplanted the increase in mesophiles (+1.91/S1, +3.33/S2, and +2.75/S3) (Figure 2).

#### Efficiency of the activated lactoperoxidase system (HT) and the N_2_-flushing (N) treatments for inhibiting bacterial growth

**Figure 3:**
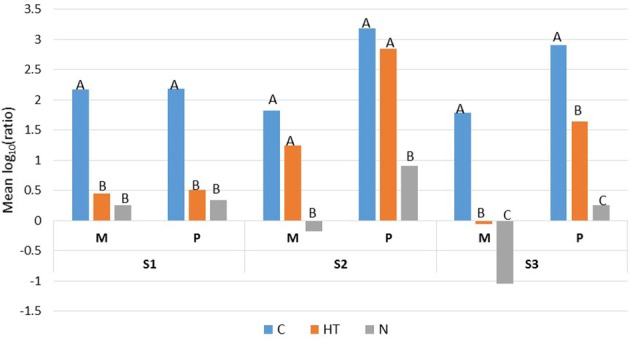
**REGWQ results describing the overall inhibitory efficiency of HT (the activated lactoperoxidase system) and N (N_**2**_ gas flushing) treatments, compared to the control (C) for the raw milk samples S1, S2, and S3 when stored at 6°C for 7 days**. Means with the same letter are not significantly different (alpha risk = 0.05).

For the three samples (S1, S2, and S3), the REGWQ test revealed that the applied treatments (HT or N) including the cold storage alone (the control condition C) promoted rather similar responses regarding the ranking of mesophiles (M) and their corresponding psychrotrophs (P) (Figure 3). The test also highlighted a sample-dependent response based on the ranking of the treatments (HT and N), which appeared to be mostly discriminatory for sample S3 because the treatments together with the control (C) were ascribed to three distinct categories (A, B, and C), unlike the samples S1 and S2, for which only two categories (A and B) were distinguished (Figure 3).

For sample S1, HT and N were evaluated as very effective and equally inhibitory as outlined by the ranking of the treatments (and grouped into category B) compared to the control (C) (ascribed to category A) (Figure 3). If HT yielded lower mean values compared to C for mesophiles and psychrotrophs, the inhibition was variable and significantly different from the control (C) only for samples S1 and S3 (Figure 3). Compared to the corresponding controls (C) and to HT, all the mean values were lowest with N; for both the mesophiles and psychrotrophs, the inhibitory effect was either equivalent to HT (for sample S1) or prevailed over HT (for samples S2 and S3). The highest inhibitory effect was recorded for mesophiles from sample S3 which was N_2_-flushed (Figure 3). For both samples S2 and S3, irrespective of the conditions (control, HT or N), the mean values for the psychrotrophs exceeded the corresponding levels for mesophiles (Figure 3).

#### Ranking of the considered antibiotics (ABs)

For all three samples, the results from the REGWQ test showed altogether rather similar mean values for mesophiles for the “no AB” condition, which ranged between 1.44 and 1.60; by contrast, higher and more variable mean values between 2.05 and 3.8 were observed for psychrotrophs (Table 2). For sample S1, the means were mostly and moderately higher for mesophiles compared to psychrotrophs outside the conditions “no AB” and “L,” whereas the S2 and S3 samples showed the highest means for psychrotrophs irrespective of the AB type (Table 2). The means for “no AB” supplanted all the conditions in which ABs were present, with two exceptions (TS-resistant mesophiles for sample S1 and G-resistant psychrotrophs for sample S2). The same order for the AB rankings (G, L, Ce and TS, from the lowest to the highest) was observed under four conditions (Table 2). The mean value was highest for TS-resistant bacteria for mesophiles and psychrotrophs in sample S1, for mesophiles in sample S2, and for psychrotrophs in sample S3 (Table 2). For both mesophiles and psychrotrophs, the ranking of the ABs was most discriminatory for sample S1, because the REGWQ test highlighted three categories (A, B, and C). For sample S2, if the mean values for the different ABs ranged between 1.70 and 3.07 for the psychrotrophs (with G-resistant psychrotrophs far above all other conditions) and between 0.67 and 1.16 for mesophiles, the statistical treatment revealed that these values were not significantly different from those of “no AB” for both population types (Table 2). For sample S3, if no significant difference was recorded with three ABs, the TS condition was not judged to be significantly different from the “no AB” condition for psychrotrophs. For mesophiles, if the Ce condition was not significantly different from that of “no AB,” then the remaining three ABs (G, TS, and L) (which all yielded negative means) were ranked at the same level. That level was distinct from the “no AB” level (Table 2). The ranking based on the “AB type” illustrated sample and AB-type dependent responses.

**Table 2:** **Ranking of the log_**10**_ (ratio) considering the antibiotics (G, Ce, L, TS) compared to the “no AB” condition by the REGWQ test**.

**Samples**	**Mesophiles**					**Psychrotrophs**				
	**Condition**	**Mean value**	Significance^*^			**Condition**	**Mean value**	Significance^*^		
**S1**	TS	1.81	A			no AB	2.05	A		
	no AB	1.60	A	B		TS	1.63	A	B	
	Ce	0.73		B	C	Ce	0.71	A	B	C
	L	0.34			C	L	0.56		B	C
	G	0.32			C	G	0.01			C
**S2**	no AB	1.54	A			G	3.07	A		
	TS	1.16	A			no AB	2.77	A		
	Ce	0.75	A			Ce	2.12	A		
	L	0.70	A			L	1.91	A		
	G	0.67	A			TS	1.70	A		
**S3**	no AB	1.44	A			no AB	3.78	A		
	Ce	0.81	A			TS	2.03	A	B	
	G	–0.20		B		Ce	1.04		B	
	TS	–0.38		B		L	0.75		B	
	L	–0.54		B		G	0.42		B	

* Means with the same letter are not significantly different (alpha risk = 0.05)

#### Trends in the log_10_ (ratio) mean values for mesophiles (M) and psychrotrophs (P)

**Figure 4:**
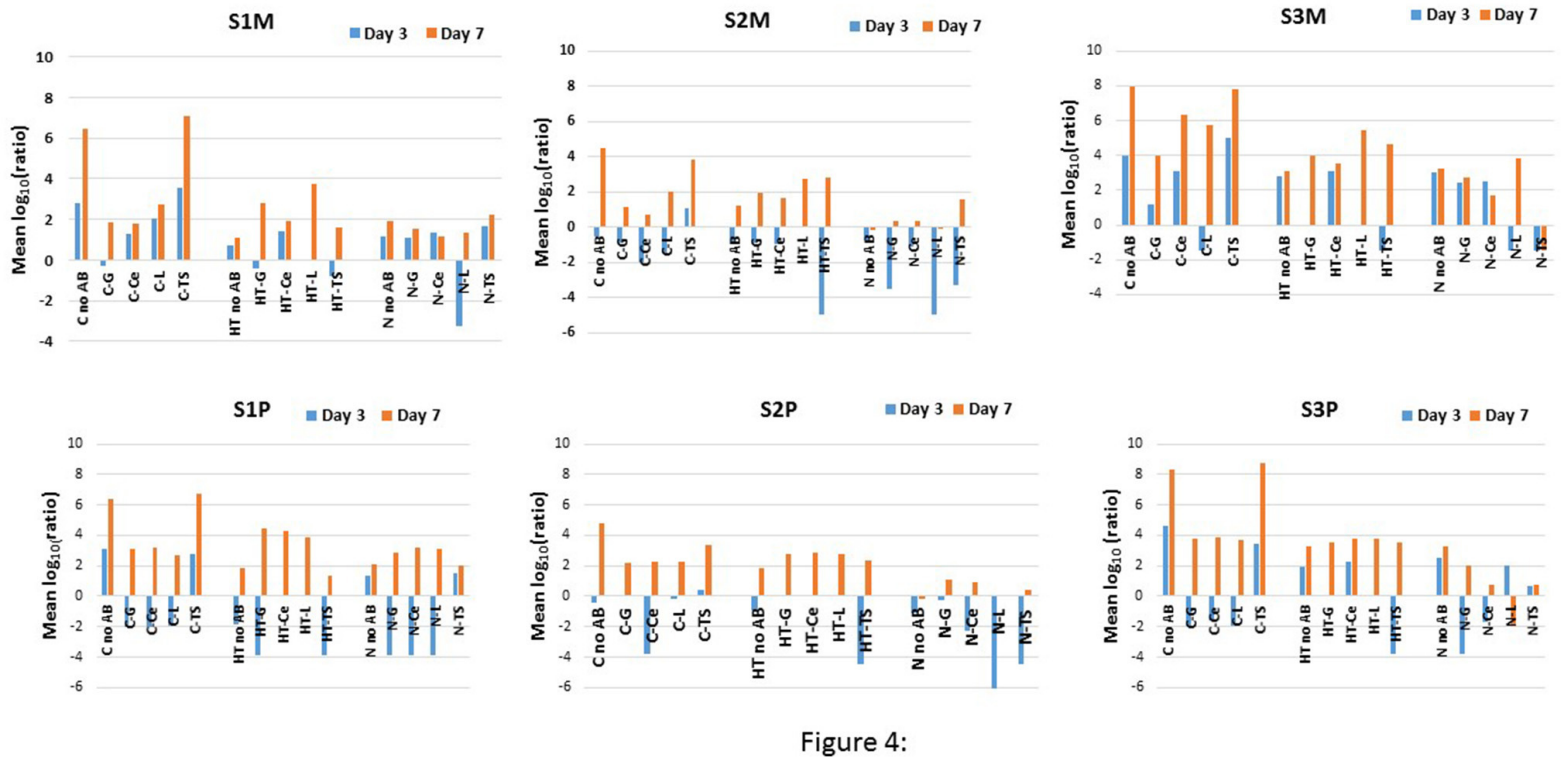
**REGWQ results showing the impact of the HT (the activated lactoperoxidase system) and N (N_**2**_ flushed milk) treatments, compared to the control (C), on the three raw milk samples S1, S2, and S3 when considering the mesophiles (S1M, S2M, and S3M) and the psychrotrophs (S1P, S2P, and S3P) for the “no AB” condition, and in the presence of the antibiotics G (gentamycin), Ce (ceftazidime), L (levofloxacin), and TS (trimethoprim-sulfamethoxazole)**.

Despite some decreases in the means at the intermediate storage time, the AR largely increased concomitantly with the increase in “total” bacterial counts which was obvious for most conditions with the exception of the TS- resistant mesophiles that were recovered from S3M with N (Figure 4). The REGWQ test revealed that the means were highest for S3M because seven conditions (five from C and two from HT) exceeded a value of 4, compared to only two conditions for S1M and one for S2M (Figure 4). All the controls (C) were characterized by high mean values with TS, which was nearly equivalent to the “no AB” condition (Figure 4). For S1M, the means were lower for HT, compared to the control (C), for the “no AB” condition and for TS, but they were higher for G and L, and moderately higher for Ce after 7 days of storage. For N, if the mean values were slightly higher for “no AB” and TS, compared to HT, all the other cases were below the levels encountered for HT or C (Figure 4). The greatest changes between the sampling points were observed for S2M, because 13 out of 15 means were negative at day 3 (with the lowest levels from HT-TS or N-L) (Figure 4). Compared to C, HT showed higher mean values for G, Ce, and L at day 7 and lower values for the conditions “no AB” and TS. By contrast, all the mean values were lower for N after 7 days under cold storage compared to the corresponding levels recorded for C but also for HT (Figure 4). The mean values were lower at day 3 in HT for S3M, compared to C for “no AB,” G and TS, and lower at day 7 for “no AB,” Ce, L, and TS. With N, the mean values at day 3 were, relative to the control (C), lower for “no AB,” Ce, and TS but higher for G and equivalent for L. However, at day 7, all the means were lowest for N compared to C, and also to HT when considering resistant bacteria (Figure 4).

The results from the REGWQ test confirmed the rise of psychrotrophs and of AR-resistant psychrotrophs under all the conditions with the exception of L-resistant bacteria for S3P with N (Figure 4). A high-growth dynamic was recorded for psychrotrophs (S1P) as many mean values shifted from negative to positive values during the storage period. After 3 days, the means were lower for HT compared to the control (C) for “no AB,” G and TS (Figure 4). The four additional storage days showed still lower means for “no AB” and for TS, but higher ones for G, Ce, and L compared to the control (C) (Figure 4). At day 3, all the means were lower for N compared to the control (C), and at day 7, still lower mean values were encountered for “no AB” and TS. The levels were moderately higher for L and equivalent for G and Ce (Figure 4). Compared to C, the means were lower for S2P in HT for “no AB” and TS and equivalent for G, Ce, and L at day 3. After 7 days of cold storage, the means were still lower for “no AB” and for TS, but higher for G, Ce, and L compared to the control (C) (Figure 4). For N, irrespective of the AB type, including the “no AB” condition, all the means were lower for S2P compared to the levels obtained for C and HT (out of one exception) (Figure 4). For S3P, HT showed lower mean values, compared to C, for “no AB” and for TS after 3 and 7 days of storage, in contrary to the results for G, Ce and L for which the means were equivalent to the levels recorded for the control (C) after 7 days (Figure 4). With one exception (L at day 3), all the mean values were lower for N compared to C at both sampling days; compared to HT, the means were equivalent for N considering “no AB” but lower for all the other conditions (Figure 4).

#### Incidence of the storage “time,” “treatment” type and antibiotic (“AB”) type factors

The contribution of the storage “time” and “treatment” type factors on the recorded effects was highly significant under all conditions; whereas the storage “time” prevailed, it also displayed great heterogeneity. For example, this factor greatly affected S1P and impacted more moderately S1M or S3P (Table 3). With the exception of S2M, the “AB” type factor had a minor and also variable contribution to the recorded effects. The ANOVA tables did not reveal any significant triple interactions between the tested storage “time,” “treatment” type and “AB” type factors. However, double interactions, that were significant at an alpha risk factor of 5%, were often recorded, even though their effects were lower compared to the effects due to the different factors that were considered individually (Table 3). For both population types from samples S1 and S2, the “treatment^*^AB” interaction was highest compared to sample S3 in which the storage “time^*^treatment” interaction dominated; the highest and nearly equivalent double interactions were recorded for S3M (Table 3).

**Table 3:** **Factors and significant double interactions that determined the recorded effects evaluated for log_**10**_ (ratio) on mesophiles (M) and psychrotrophs (P) for raw milk samples S1, S2, and S3**.

**Sample - population**	***F* value for the factors**	Pr > F^*^	**Double interactions**	Pr > F^*^
S1M	Time F 51.46	<0.0001	Treatment^*^AB F 8.64	<0.0001
	Treatment F 23.88	<0.0001	Time^*^AB F 3.15	0.0194
	AB F 6.31	0.0002		
S1P	Time F 198.49	<0.0001	Treatment^*^AB F 7.63	<0.0001
	Treatment F 13.21	<0.0001	Time^*^AB F 5.51	0.0007
	AB F 4.69	0.0021		
S2M	Time F 134.56	<0.0001	Treatment^*^AB F 4.71	0.0001
	Treatment F 17.24	<0.0001	Time^*^AB F 3.29	0.0159
S2P	Time F 120.87	<0.0001	Treatment^*^AB F 4.14	0.0005
	Treatment F 19.56	<0.0001	Time^*^AB F 2.16	0.0826
	AB F 2.61	0.0431		
S3M	Time F 117.89	<0.0001	Time^*^treatment F12.88	<0.0001
	Treatment F 43.06	<0.0001	Time^*^AB F 11.80	<0.0001
	AB F 9.22	<0.0001	Treatment^*^AB F 10.87	<0.0001
S3P	Time F 45.45	<0.0001	Time^*^treatment F 6.21	0.0033
	Treatment F 10.25	0.0001	Treatment^*^AB F 3.77	0.0010
	AB F 6.44	0.0002		

* *p value associated with the F statistic*.

## Discussion

The trend in AR over time was evaluated here for mesophilic and psychrotrophic bacteria that were present in three bovine raw milk samples, which were subjected to a 7-day cold storage period at 6°C. Single cold storage, which counted as the control (C), was combined with either the activated lactoperoxidase system (HT) or with a continuous N_2_ gas flushing treatment (N). At the initial stage of the analyses, because the “total bacterial counts” (“no AB” condition) ranged between 2.86 and 3.57 log-units (Figure 1), the raw milk samples can be considered to be of very good bacteriological quality (Anonymous, 2004). This point is also corroborated by the initial pH values (Table 1) that are indicative of fresh raw milk (Tetra Pak, 2003).

Single cold storage largely promoted bacterial growth in a sample-dependent matter (Figures 1–4). The three samples were characterized by initial psychrotroph/mesophile ratios of 11.3, 4.5, and 1.3% for samples S1, S2, and S3, respectively, which suggests a shorter cold storage period prior to the start of the analyses for samples S3 and S2 compared to that of sample S1. The idea, that sample S1 probably underwent a longer cold storage prior to analyses is also corroborated by the observation of the growth kinetic for mesophiles (S1M) and especially psychrotrophs (S1P), which was highest for the “no AB” condition for sample S1 because the counts exceeded 10^5^ cfu/ml already after 3 days of storage at 6°C (Figure 1). By contrast, sample S2 showed the slowest growth kinetics for both mesophiles (S2M) and psychrotrophs (S2P) because the counts were only slightly above 3 log-units for both population types after 3 days (Figure 1); however, after 7 days, both the mesophiles and psychrotrophs reached approximately 8 or 9 log-units cfu/ml and were nearly equivalent in all three samples (Figure 1). If an altogether similar growth dynamic characterized the psychrotrophs and mesophiles from sample S1 [as estimated from the mean log_10_ (ratio) values of approximately 2.2 for both population types (Figure 3), which further tends to confirm a longer cold storage period for S1 prior to the analyses], it was distinct from the results for samples S2 and S3 in which the overall growth dynamic for the psychrotrophs was most vigorous [with mean log_10_ (ratio) values of 3.19 and 2.91, respectively] compared to the mesophiles [with mean log_10_ (ratio) values of 1.83 and 1.79, respectively; Figure 3]. After 7 days in cold storage, the control milk samples (C) were spoiled, exhibited the lowest pH values and emitted bad odors, whereas the pH values for HT- treated milk or N_2_-flushed samples were not greatly altered (Table 1), and were in line with previous observations (Gaya et al., 1991; Dechemi et al., 2005; Munsch-Alatossava et al., 2010a).

Indisputably, the rise in bacteria in the raw milk during cold storage corresponded to an increase in resistant mesophilic and psychrotrophic bacterial populations (Figures 1–4), confirming earlier observations; shifts in the AR profiles over time, together with very high TS- resistance levels at the end of the storage period were also previously observed for cold-stored raw milk samples (Munsch-Alatossava et al., 2012a,b). Moreover, the highest AR increase for psychrotrophs compared to mesophiles as noted here for samples S2 and S3 (Figures 1, 4) is also in line with previous observations which showed that AR, that was evaluated from psychrotrophs, supplanted AR from the corresponding mesophiles in cold-stored raw milk (Munsch-Alatossava et al., 2012a); this point also highlights the difficulty of judging the quality of raw milk with the sole mesophilic bacteriological standard, in which psychrotrophs may be partially overlooked due to their growth optima as not all psychrotrophs grow under mesophilic conditions.

The ranking of the ABs (Table 2) occurred in accordance with the frequency of AB usage (Kools et al., 2008).

The activation of the lactoperoxidase (an oxidoreductase that is naturally present in bovine raw milk), which is recommended by the FAO to preserve raw milk in developing countries, triggers a bacteriostatic type of action against Gram-positive bacteria such as streptococci or lactobacilli and a bactericidal type of action for Gram-negative such as coliforms or *Pseudomonas* spp. (Björck et al., 1975; Wolfson and Sumner, 1993; FAO, 2005; Seifu et al., 2005). The temperature-dependent inhibitory effect allows for an extension of the keeping quality for raw milk by 11–12 h at 25°C or by 5–6 days at 4°C (FAO, 2005). The described inhibitory effect with HT was especially noticeable here for samples S1 and S3, where, for “no AB,” both the mesophilic and psychrotrophic counts were below the counts from the control (C) at day 3, and still below 3 × 10^5^ cfu/ml after 7 days (Figure 1), which is consistent with the literature (FAO, 2005). A bactericidal type of effect due to HT could be suspected for psychrotrophs from S1 (S1P) (Figures 1, 4).

For all three samples, the activated lactoperoxidase system revealed two effects that succeeded one another over time. Specifically, based on the “total counts” level, the initial storage phase until day 3 showed an AB type-dependent AR level; but at day 7, even though the “total counts” (for “no AB”) were still below or approximately 3 × 10^5^ cfu/ml (for mesophiles) for all the conditions, both the psychrotrophic and mesophilic bacterial types resistant to G, Ce, L and TS were often plateauing at approximately the same level, contrary to that of the control (C) or to the N_2_-flushed milk samples (N) (Figure 1). Surprisingly, the resistant bacterial counts enumerated under the activated lactoperoxidase system (HT) could even exceed the resistant colony levels encountered from the corresponding controls (C) as noticeable for mesophiles in sample S2 (Figure 1). All the preceding observations highlight an altogether greater growth dynamic under the activated lactoperoxidase system combined with cold storage (HT), when compared to the single cold storage (C), which resulted in a selection of bacterial populations that were recovered at similar levels from different AB platings, implying that these bacterial types carry AB multi-resistance features.

The continuous N_2_ gas flushing kept both the mesophilic and psychrotrophic “total” counts (condition “no AB”) below 10^5^ cfu/ml over 7 days for all three samples (Figure 1) as observed previously (Munsch-Alatossava et al., 2010a; Gschwendtner et al., 2016). The recorded inhibitory effects due to N_2_ gas flushing were also more “sample or initial microbiota than initial total bacterial level”-dependent (Figure 1); this point is well illustrated for S2M and S3M. Compared to sample S2, sample S3 presented the lowest psychrotrophic bacterial level at the initial stage and showed the highest sensitivity to N_2_-flushing with a 5-fold lower mean for S3, despite the fact that both samples presented approximately similar initial bacterial counts (Figures 1, 3). Notably, the study showed that N_2_ flushing was always efficient at controlling bacterial growth and the increases in AR as the mean log_10_ (ratio) values were judged to be significantly different for N compared to C for the three samples, or from HT for two samples (Figure 3); additionally, the means were mostly lowest for the N_2_-flushed milk samples irrespective of the AB type (Figure 4). As frequently reported, *Pseudomonas* and *Acinetobacter* are key spoilers of raw milk (Cousin, 1982; Wiedmann et al., 2000; Chambers, 2002; Munsch-Alatossava and Alatossava, 2006; Rasolofo et al., 2010); isolates from both genera, that were retrieved from raw milk samples, also exhibited AB multiresistant traits (Munsch-Alatossava and Alatossava, 2007). Both of these genera host nosocomial pathogens and are well known for their intrinsic resistance to ABs together with a remarkable ability to acquire genes encoding resistance determinants (Bonomo and Szabo, 2006). The results from the present cultivation-dependent approach, together with the NGS based study which revealed that both *Pseudomonas* and *Acinetobacter* were negatively affected by the N_2_ flushing (Gschwendtner et al., 2016), are further heightening the potential of the N_2_ gas-based treatment.

For the first time, the inhibitory effect of N_2_ was “quantified” by the log_10_ (ratio) variable, which reflects the growth trend over a cold storage period of 7 days. Compared to the single cold storage, N_2_ gas flushing was here, 81, 97, and 693-fold more efficient in controlling the growth of mesophiles in raw milk for samples S1, S2 and S3, respectively. The comparative analyses of sample S3 on one side and of S1 on the other side suggest that an earlier gas application is most appropriate for preventing a massive bacterial increase and a simultaneous increase in AR (Figure 3). Some conditions yielded negative mean values (Figures 3, 4), which suggests that N_2_ gas flushing could also trigger a bactericidal-type of effect in addition to exhibiting an overall bacteriostatic type of effect. To date, no clear bactericidal action was highlighted out of the observation that for *Bacillus weihenstephanensis* (a Gram-positive representative found in pasteurized milk), the cells were considerably damaged according to electron microscopic observations that were taken following the N_2_ gas flushing treatment (Lechner et al., 1998; Munsch-Alatossava et al., 2013; Munsch-Alatossava and Alatossava, 2014).

Taken together, the trends in the mean values from both treatments (HT and N), including the cold storage alone (C), illustrated the sample or bacterial population type-, treatment type-, antibiotic type-, and time-dependent responses. As expected, the bacterial populations were best controlled at day 3 compared to day 7 for both treatments (Figures 1, 2).

It was previously observed that the effectiveness of N_2_ in controlling bacterial growth in raw milk samples stored at 15 or 25°C was somehow equivalent to that of HT (Munsch-Alatossava et al., 2016); surprisingly, at 6°C, the N_2_-based treatments seemed to be more efficient at inhibiting bacterial growth and also at tackling AR in bacterial populations compared to HT (Figures 1–4). At 6°C, the “storage time” factor was preponderant over the “treatment type” under all the conditions (Table 3), in contrast to the study at 15/25°C in which the “treatment type or condition“ factor seemed to be the major determinant of the inhibitory effects for 4 of 7 samples (Munsch-Alatossava et al., 2016). A notable difference between HT and N relied on the observation that at sampling day 3, the activated lactoperoxidase system (HT) reduced TS-resistant bacterial levels to non-detectable levels under four conditions (S1P, S2M, S3M, and S3P) (Figure 1) whereas N experienced a reduction in the L-resistant bacterial types under four conditions (S1M, S2M, S2P, and S3M) (Figure 1).

Because a “plateau situation” was observed less frequently with N_2_ compared to HT, it can be reasonably assumed that both treatments do not target the same bacterial types and that the mode of action of the two treatments is different. As shown in Figure 1, the N_2_ gas treatment seems to trigger more moderate changes compared to HT in which large dynamic changes such as a 10^5^-fold increase for TS-resistant bacteria could occur within 4 days.

We hypothesize that the activation of the lactoperoxidase system as an enzymatic type of reaction occurs rapidly and creates a “biological emptiness” that can be exploited by certain bacterial types that clearly dominated at the end of the cold storage phase and were characterized by the ability to express resistance to four classes of antibiotics simultaneously (Figures 1–3). Cold storage promotes an increase of Gram-negative bacteria in raw milk (Chambers, 2002); the opportune action of HT that reportedly exerts primarily bactericidal action on Gram-negative representatives is therefore most appropriate for reducing the bacterial load. For all three samples considered here, the activated lactoperoxidase system clearly favored the growth of multi-resistant bacterial populations (Figures 1, 4), which suggests that the activated lactoperoxidase system may not confer full benefits to all raw milk samples; whether initial good intentions are diverted by bacterial activities requires further clarifications.

Microorganisms, which are present in freshly drawn raw milk, are subjected to a cold shock (from 37°C to 3–4°C). After that, cold storage promotes considerable changes in bacterial populations with a reduction in bacterial diversity, including the decay of some genera (Lafarge et al., 2004; Raats et al., 2011; Munsch-Alatossava et al., 2012b; Gschwendtner et al., 2016).

The graphs in Figure 1 showed that the N treatment seems to preserve the bacterial diversity better during cold storage because the growth trend, under the different conditions, mostly reflects a “shift of a certain initial AR pattern” over time. The point that N_2_ gas alleviated the loss of bacterial diversity during cold storage was already highlighted by the NGS approach (Gschwendtner et al., 2016); we hypothesize that by better preserving bacterial diversity during cold storage, the N_2_ gas flushing may better prevent the occurrence of a “favorable to a few” niche and consequently better prevent the rise of antibiotic multi-resistance in raw milk.

Bacterial genomes carry between 1 and 15 ribosomal RNA operons (rrn) (Klappenbach et al., 2000; Větrovsky and Baldrian, 2013). Pal et al. (2016) found a relative abundance of AR genes at 0.035 copies per 16S rRNA gene by investigating the human, animal and environmental resistomes; by considering a 10^5^ cfu/ml raw milk sample and assuming an average of seven ribosomal RNA operons per bacterial cell, this level would correspond to as many as 24,500 AR genes in one milliliter of raw milk.

Further studies are needed to examine whether “most fresh raw milk” is under conditions that call for N_2_ gas flushing to maximize the efficiency of blocking the increase in both “total counts” and in AR bacteria in raw milk during its cold storage.

## Conclusions

Bovine raw milk as a highly perishable food material is the target of many different bacterial types with both spoilage and AR features. Here, N_2_ gas flushing (N) kept the “total counts” of bacteria below 3 × 10^5^ cfu/ml for three samples over 7 days of storage at 6°C, but also demonstrated real potential in preventing high increases in mesophiles or psychrotrophs carrying AR or multi-resistant features. The efficiency, evaluated by the log_10_ (ratio), was maximal for the milk sample that carried low mesophilic counts and the lowest psychrotrophic initial counts. Compared to the activated lactoperoxidase system (HT), the quantified inhibitory effect revealed equal or superior efficiency for N; if the bacterial levels were also kept below the 3 × 10^5^ cfu/ml limit for HT, the treatment combined with cold storage was selective for highly multi-resistant populations in all three samples. By contrast, if N_2_ gas flushing conferred benefits in terms of keeping quality of raw milk by maintaining bacterial “total counts” under the 3 × 10^5^ cfu/ml limit, it did not favor that frequent and massive proliferation of antibiotic multi-resistant bacteria.

## Author contributions

PMA and TA conceived and designed the experiments; PMA and SJ performed the experiments; JPG analyzed the data; TA and JPG contributed reagents/materials/analysis tools; PMA, TA, and JPG wrote the paper. All authors approved the final version.

### Conflict of interest statement

The authors declare that the research was conducted in the absence of any commercial or financial relationships that could be construed as a potential conflict of interest.

## Abbreviations

S1, S2, S3: raw milk samples
M: mesophiles
P: psychrotrophs
C: control
HT: activated lactoperoxidase system
N: N_2_ gas flushing
AR: antibiotic resistance
AB: antibiotic
G: gentamycin
Ce: ceftazidime
L: levofloxacin
TS: trimethoprim-sulfamethoxazole.
